# Supplementary material for: Articular surface interactions distinguish dinosaurian locomotor joint poses
Source: Nat Commun. 2024 Feb 16;15:854. doi: 10.1038/s41467-024-44832-z (PMC10873393; doi:10.1038/s41467-024-44832-z)
Supplement: Supplementary file 1 — Supplementary Information [file 41467_2024_44832_MOESM1_ESM.pdf]

Supplementary Information

Articular surface interactions distinguish dinosaurian locomotor joint poses

Armita R. Manafzadeh[1,2,3,4*], Stephen M. Gatesy[5], Bhart-Anjan S. Bhullar[2,3]

[1]Yale Institute for Biospheric Studies, Yale University, New Haven, CT 06520
[2]Department of Earth & Planetary Sciences, Yale University, New Haven, CT 06520
[3]Yale Peabody Museum of Natural History, New Haven, CT 06520
[4]Department of Mechanical Engineering & Materials Science, Yale University, New Haven, CT 06520
[5]Department of Ecology, Evolution, and Organismal Biology, Brown University, Providence, RI, 02912
*Email for correspondence: armita.manafzadeh@yale.edu

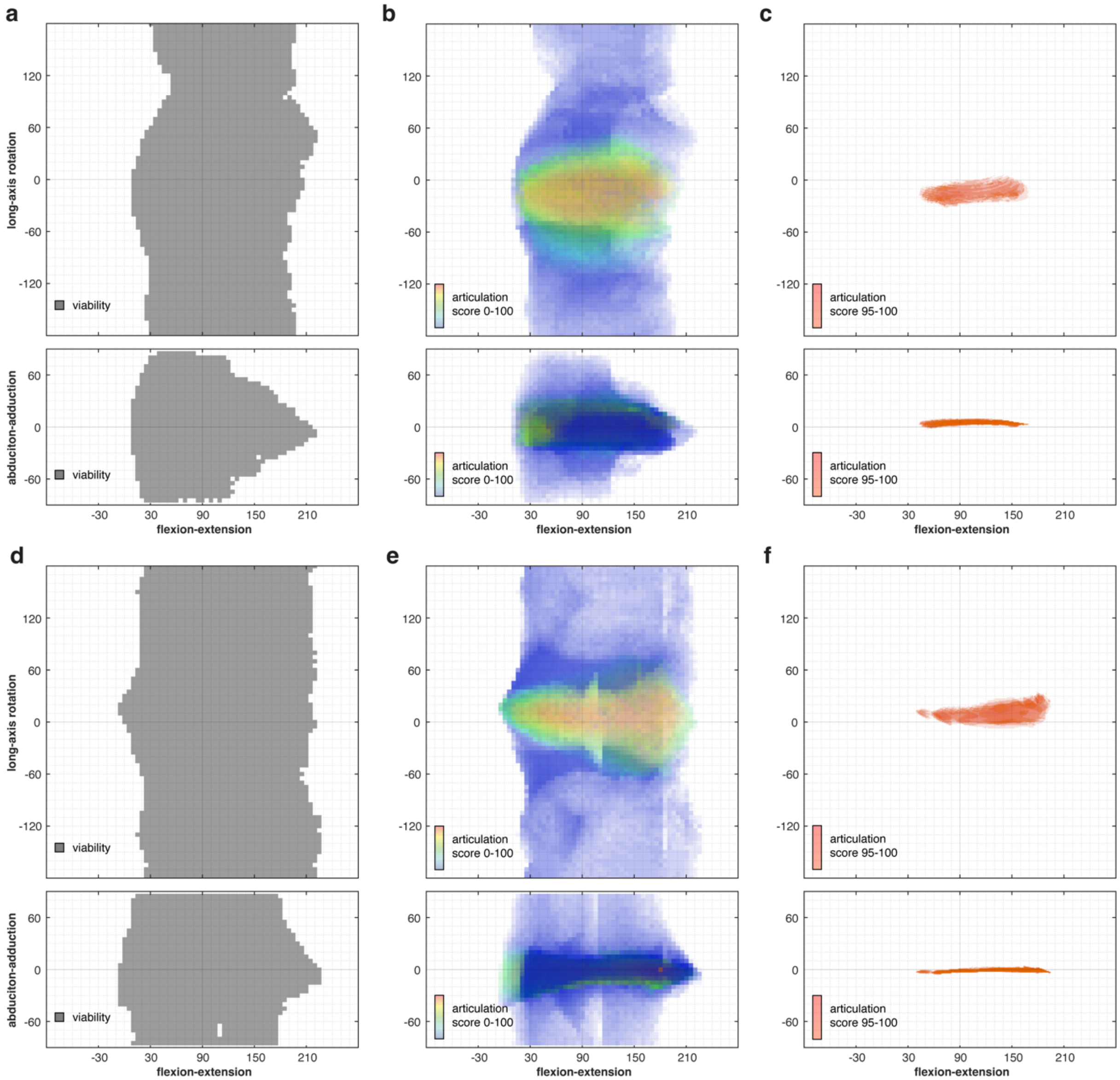

Supplementary Figure 1. Additional views of extant dinosaurian ankle joint articulation analysis results. (**a**) Helmeted Guineafowl viability based on bone-bone contact at five-degree resolution. (**b**) Helmeted Guineafowl articulation scores at five-degree resolution. (**c**) Top Helmeted Guineafowl articulation scores at one-degree resolution. (**d**) Common Emu viability based on bone-bone contact at five-degree resolution. (**e**) Common Emu articulation scores at five-degree resolution. (**f**) Top Common Emu articulation scores at one-degree resolution.

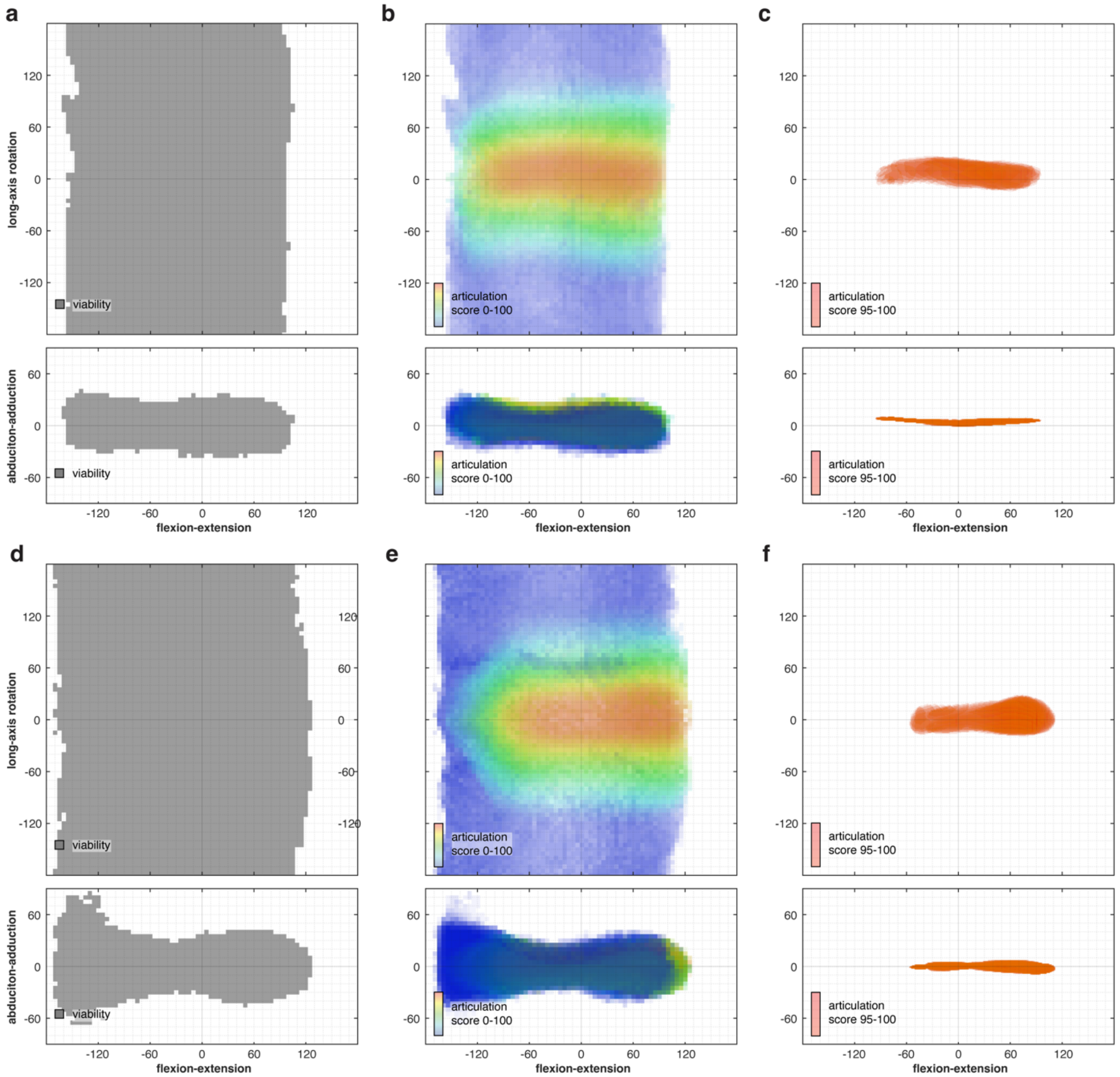

Supplementary Figure 2. Additional views of extant dinosaurian metatarsophalangeal (MTP) joint articulation analysis results. (**a**) Helmeted Guineafowl viability based on bone-bone contact at five-degree resolution. (**b**) Helmeted Guineafowl articulation scores at five-degree resolution. (**c**) Top Helmeted Guineafowl articulation scores at one-degree resolution. (**d**) Common Emu viability based on bone-bone contact at five-degree resolution. (**e**) Common Emu articulation scores at five-degree resolution. (**f**) Top Common Emu articulation scores at one-degree resolution.

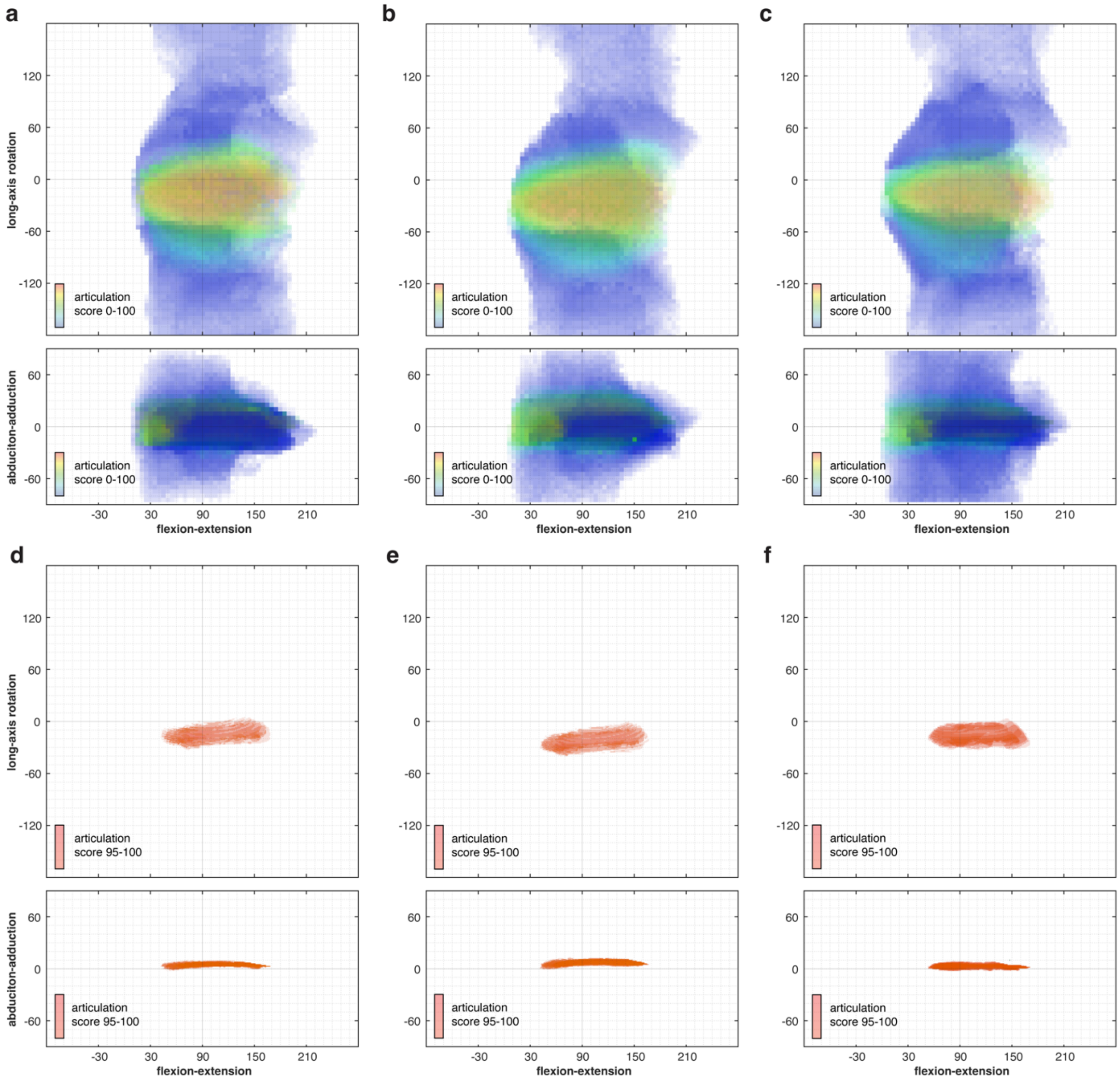

Supplementary Figure 3. Sensitivity analysis of ankle joint articulation results using three guineafowl individuals. As discussed in the Methods, articulation analyses were conducted on the ankle joints of three guineafowl individuals to determine the sensitivity of results to intraspecific morphological variation. Results from the guineafowl individual figured in the main text, displayed at five-degree angular resolution in (**a**) and one-degree angular resolution in (**d**); a second individual, displayed at five-degree angular resolution in (**b**) and one-degree angular resolution in (**e**); and a third individual, displayed at five-degree angular resolution in (**c**) and one-degree angular resolution in (**f**), are grossly similar, especially within the highest-scoring region.

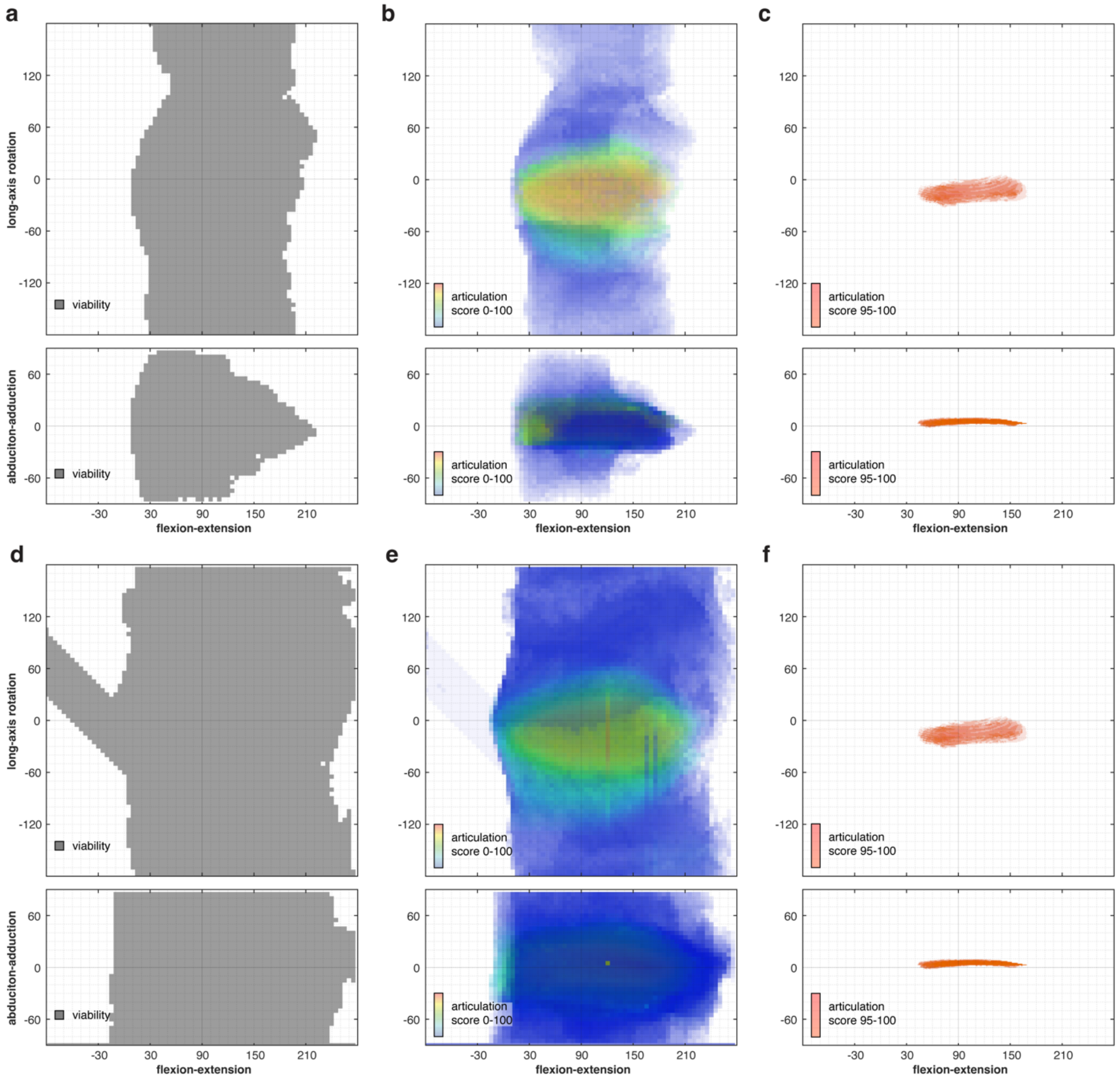

Supplementary Figure 4. Sensitivity analysis of ankle joint articulation results using increased translational allowance. As discussed in the Methods, translational sensitivity analysis by allowing 1,331 translational combinations over a larger range (two additional increments of equal size at each end of the X, Y, and Z translation ranges). Results from both the translational allowance throughout this paper, displayed at five-degree angular resolution in (**a**) and (**b**) and one-degree angular resolution in (**c**), and an increased translational allowance, displayed at five-degree angular resolution in (**d**) and (**e**) and one-degree angular resolution in (**f**), demonstrate that although increasing translational allowance increases the region of pose space coded as viable (and therefore the region of pose space receiving articulation scores), the highest-scoring region of pose space remains identical.

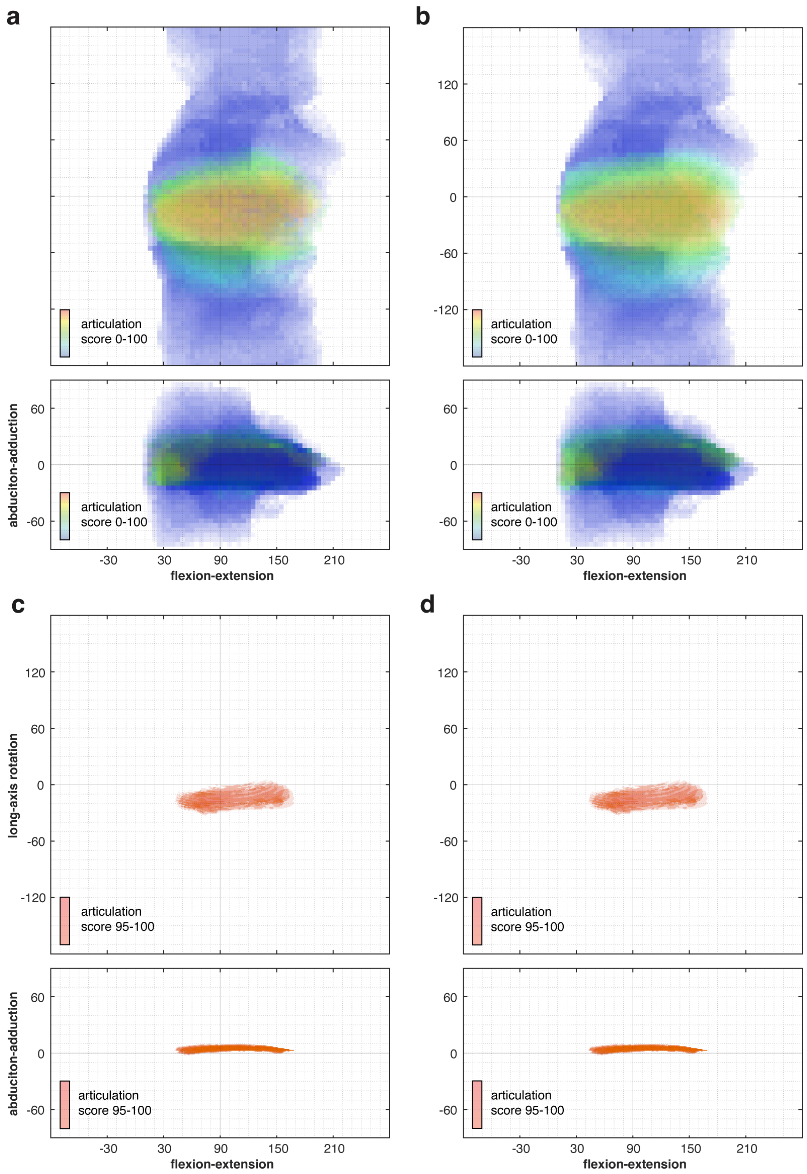

Supplementary Figure 5. Sensitivity analysis of guineafowl ankle joint articulation results using an alternative score formula. As discussed in the Methods, sensitivity to articulation score formula was evaluated by analyzing a guineafowl ankle at the original rotational and translational resolution and weighting congruence by single-condyle overlap rather than full-joint overlap. Results from both the articulation score formula implemented throughout this paper, displayed at five-degree angular resolution in (**a**) and one-degree angular resolution in (**c**), and an alternative formula that instead weights each condyle's congruence by only its own overlap (rather than average overlap; see Methods), displayed at five-degree angular resolution in (**b**) and one-degree angular resolution in (**d**), are grossly similar.

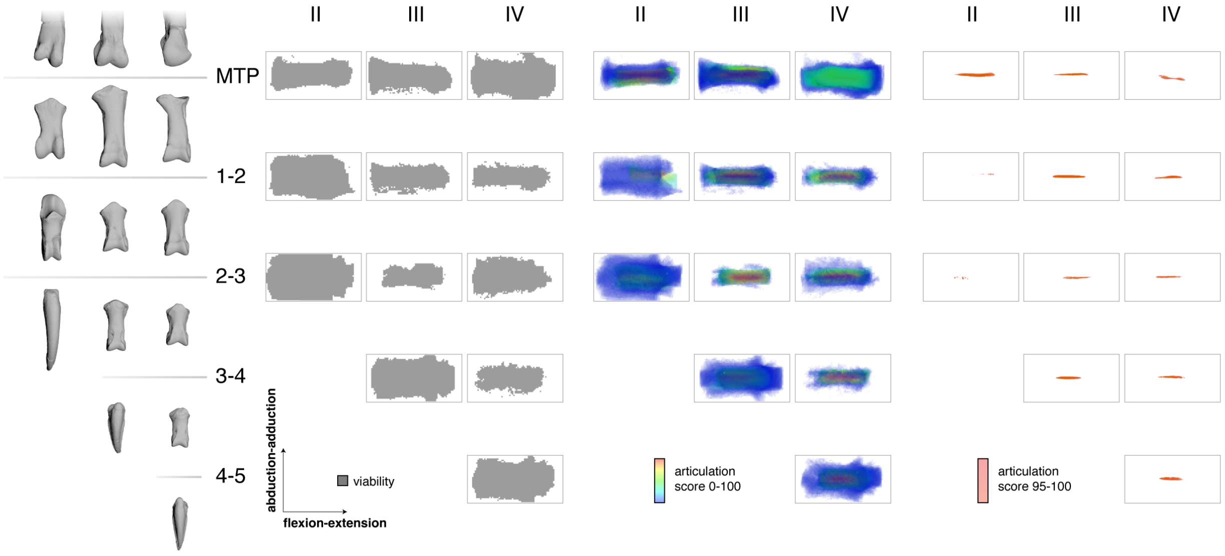

Supplementary Figure 6. Additional views of extinct dinosaurian (*Deinonychus*) pedal joint articulation analysis results. Pedal elements of YPM 5205 in dorsal view, with results of metatarsophalangeal and interphalangeal articulation analyses colored by viability based on bone-bone contact alone (left) and articulation score (middle and right), analyzed at five-degree (left and middle) and one-degree (right) angular resolution. Abduction-adduction axes from -90 to 90 degrees and flexion-extension axes from -180 to 180 degrees in all graphs. Note the variation in articulation score distribution from 0-100 in the abduction-adduction dimension across joints, particularly the broader abduction-adduction extent of mid-range (green-toned) articulation scores at the MTP IV joint. See also Fig. 3 and Supplementary Fig. 7.

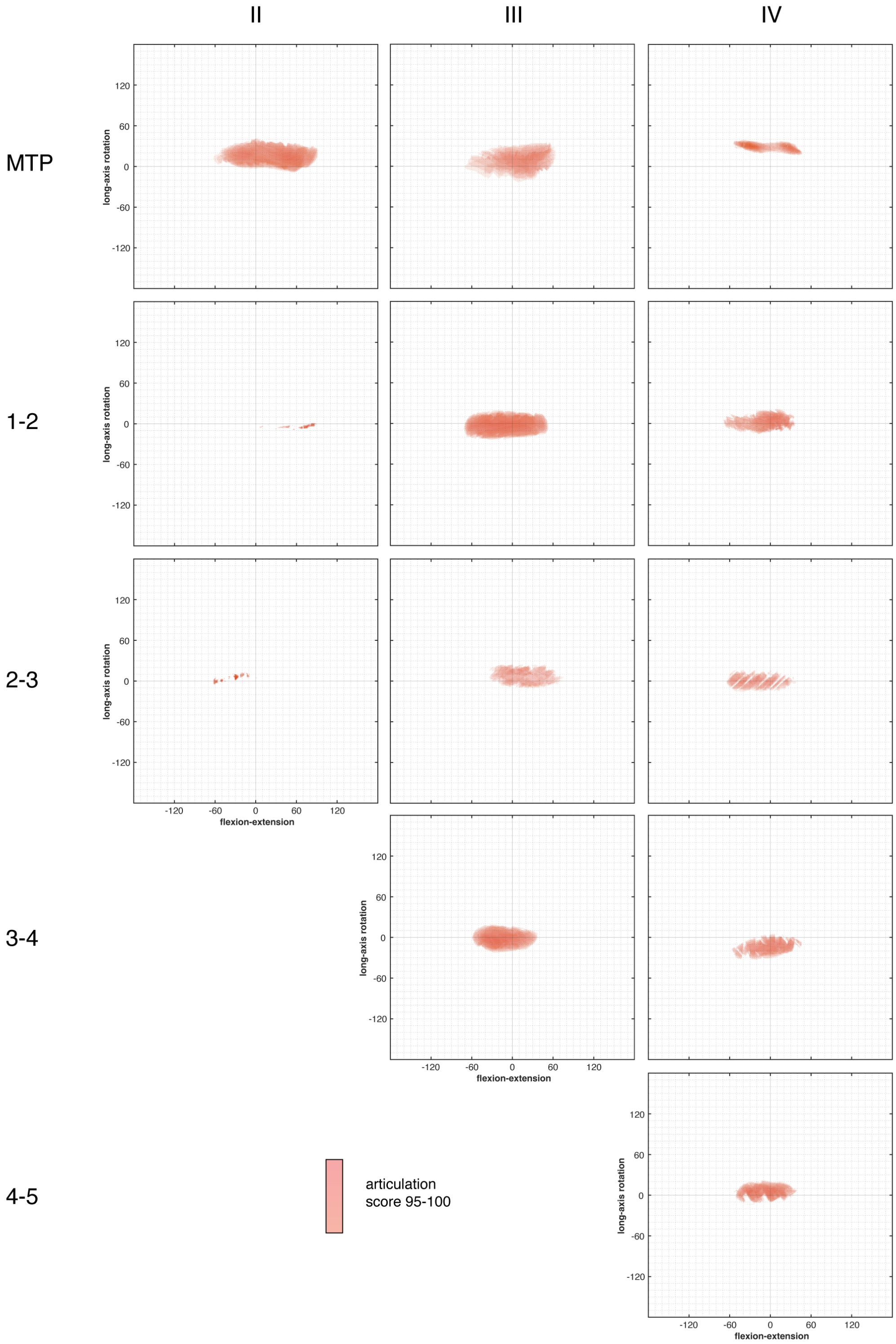

Supplementary Fig. 7. Detailed view of extinct dinosaurian (*Deinonychus*) pedal joint articulation analysis results. Results of metatarsophalangeal and interphalangeal articulation analyses colored by articulation score, analyzed at one-degree angular resolution. See also Fig. 3 and Supplementary Fig. 6, 8.

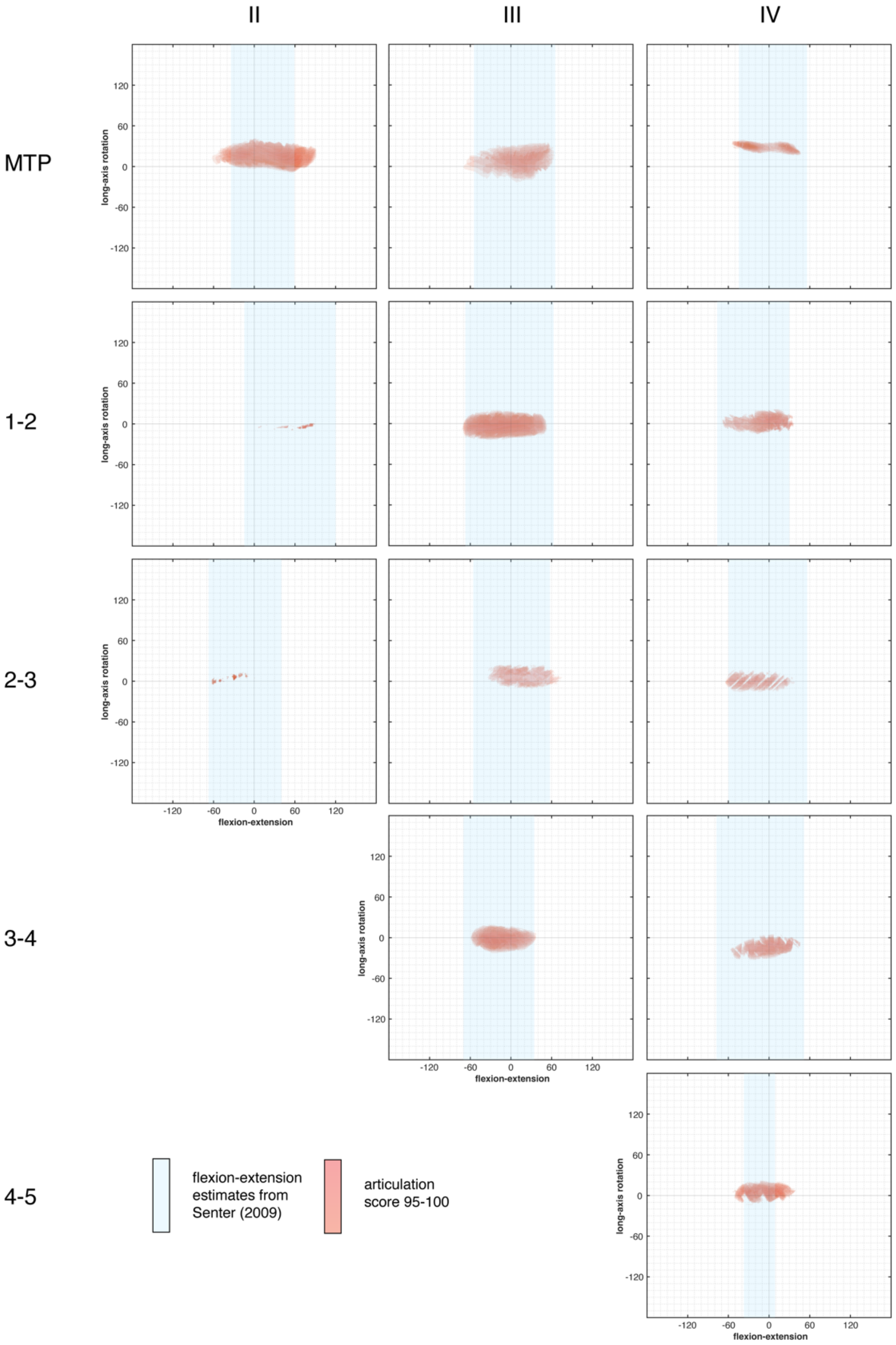

Supplementary Fig. 8. Comparison of this study's results with those of Senter. Results of metatarsophalangeal and interphalangeal articulation analyses colored by articulation score, analyzed at one-degree angular resolution, compared against the flexion-extension range of motion estimates provided by Senter (2009). See also Fig. 3 and Supplementary Fig. 6-7. (Senter, P. (2009). Pedal function in deinonychosaurs (Dinosauria: Theropoda): a comparative study. *Bulletin of the Gunma Museum of Natural History*, *13*, 1-14.)

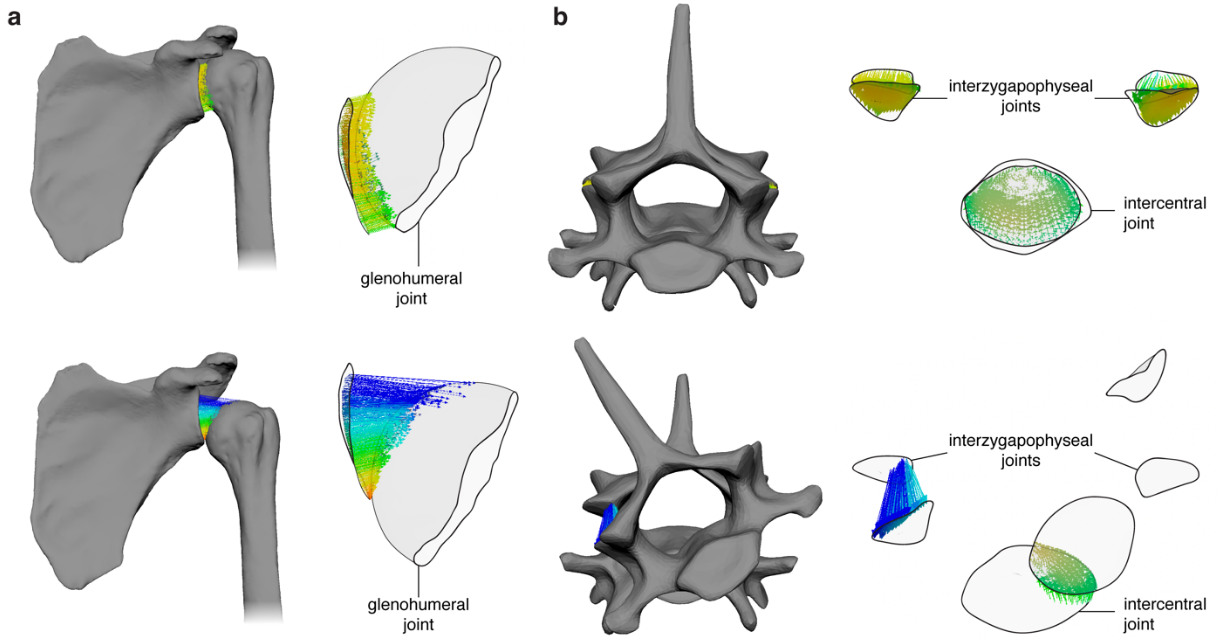

Supplementary Fig. 9. Preliminary demonstration of generalizability of articular raycasting. Raycasts conducted on a (**a**) human glenohumeral joint and (**b**) feline intervertebral joint suggest that articular raycasting can be used in future studies to investigate articular quality to differentiate likely higher- (top) from lower-scoring (bottom) six-degree-of-freedom configurations based on overlap, symmetry, and congruence. Example articulation subscores for the configurations presented here are as follows for higher- (top) vs. lower-scoring (bottom) configurations: glenohumeral overlap: 1.000 vs. 0.816; glenohumeral congruence: 0.741 vs. 0.512; interzygapophyseal overlap: 0.725 and 0.648 vs. 0.299 and 0.000; intercentral overlap: 0.658 vs. 0.281; interzygapophyseal congruence: 0.825 and 0.818 vs. 0.631 and 0.000; intercentral congruence: 0.805 vs. 0.759. We caution that drawing inferences based on articulation analysis for these joints will require full sampling of rotational pose space as well as comparison with *in vivo* kinematic data specific to the behavior or behaviors of interest. Human scapular and humeral mesh models obtained from the National Institutes of Health 3D Portal (NIH3D; https://3d.nih.gov/entries/3DPX-000387), cat vertebrae obtained from the Harvard Dataverse (https://doi.org/10.7910/DVN/XP3JVZ).

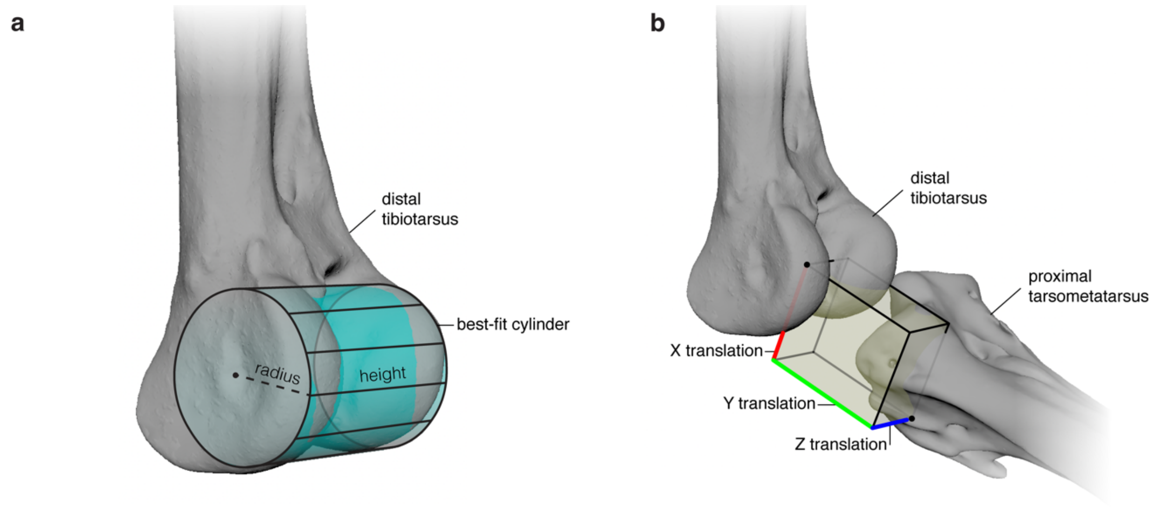

Supplementary Fig. 10. Selection of translations for joint configuration sampling. Translations at each joint are selected based on multiples of **(a)** a cylinder fit to the distal condyles of the proximal bone, as described in Supplementary Data 1, and implemented based on **(b)** the prism-based hinge joint translation convention outlined by Manafzadeh & Gatesy (2021).

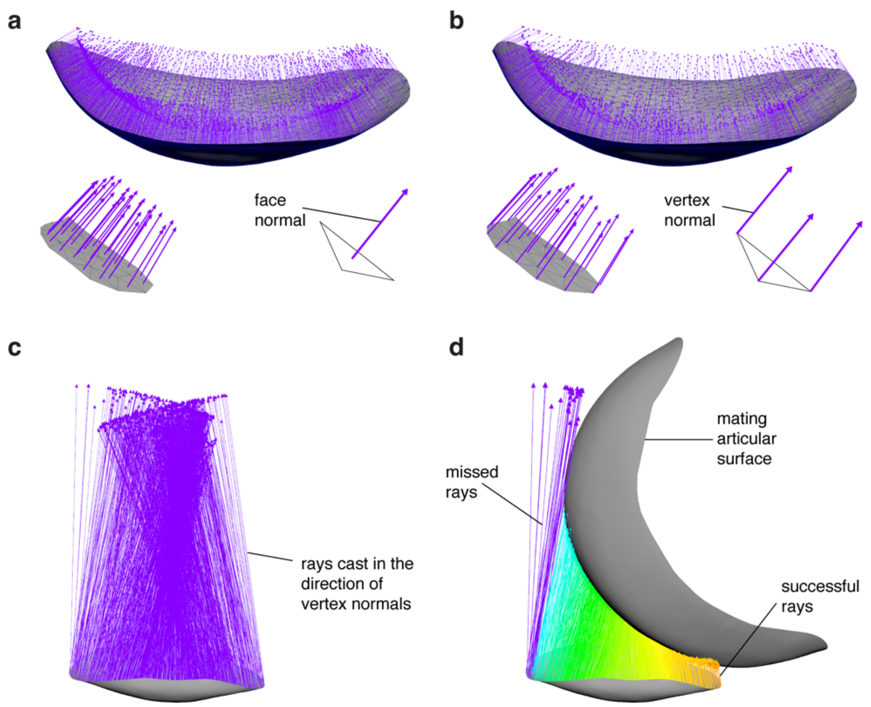

Supplementary Figure 11. Fundamentals of articular raycasting. (**a**) Any single face of a 3-D mesh has a 3-D orientation in space, and a vector drawn orthogonally to that face based on its orientation is called a "face normal". (**b**) Averaging the face normals of all faces surrounding a vertex yields a "vertex normal" for each vertex of the mesh. (**c**) To conduct an articular raycast, we cast rays in the direction of all of the vertex normals of one articular surface. (**d**) We then checked how many of those rays were oriented such that they hit the mating articular surface, forming vectors of a specific length connecting both surfaces (colored orange through blue), and how many rays instead shot past the mating articular surface, missing it entirely (purple). See Methods.

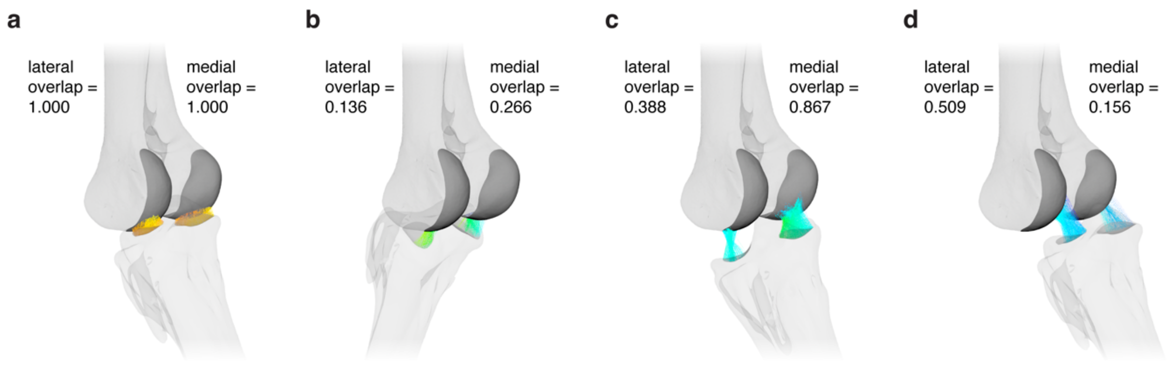

Supplementary Figure 12. Measurement of articular surface overlap. (**a**) A high-scoring joint pose with perfect overlap on both medial and lateral sides. Reduced overlap can result from various configuration changes, such as (**b**) articular surfaces flexing or extending past each other's borders, (**c**) articular surfaces long-axis rotating past each other's borders, or (**d**) articular surfaces translating past each other's borders. (Rays colored by length; missed rays are not shown.)

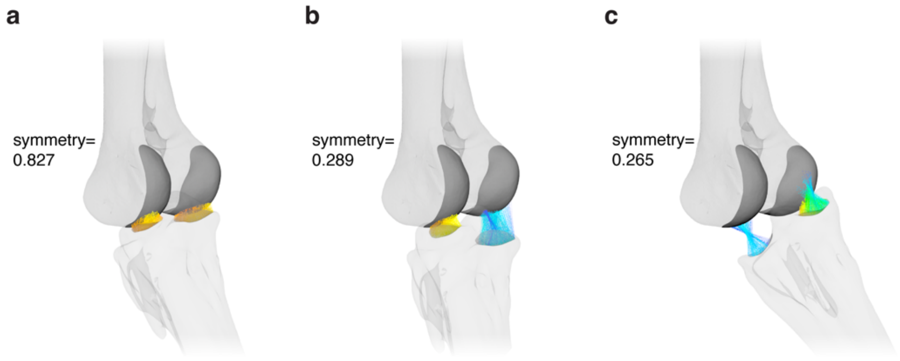

Supplementary Figure 13. Measurement of articular surface symmetry. (**a**) A high-scoring joint pose with high symmetry in average ray length between medial and lateral sides. Reduced symmetry can result from various configuration changes, such as (**b**) abduction or adduction of the joint creating uneven average ray length between joint halves, or (**c**) long-axis rotation of the joint creating uneven average ray length between joint halves. (Rays colored by length; missed rays are not shown.)

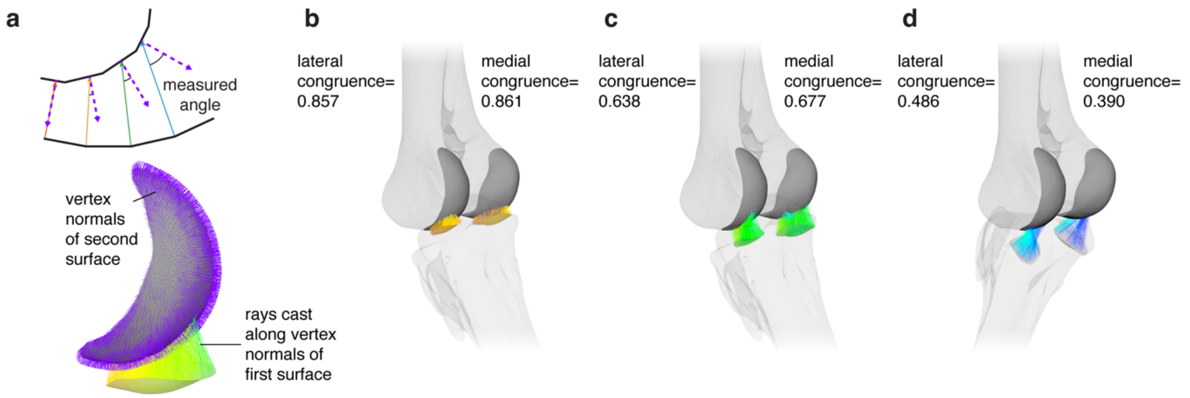

Supplementary Figure 14. Measurement of articular surface congruence. (**a**) Articular surface congruence is measured based on angles between rays cast along the vertex normals of one surface, and the normals of the location they hit on the other surface (low average angles result in a high congruence subscore). (**b**) A high-scoring joint pose with high congruence subscores on both medial and lateral sides. Reduced overlap can result from various configuration changes, such as (**c**) changes in joint spacing, or (**d**) certain six-degree-of-freedom combinations of rotation and translation. (Rays colored by length; missed rays are not shown.)

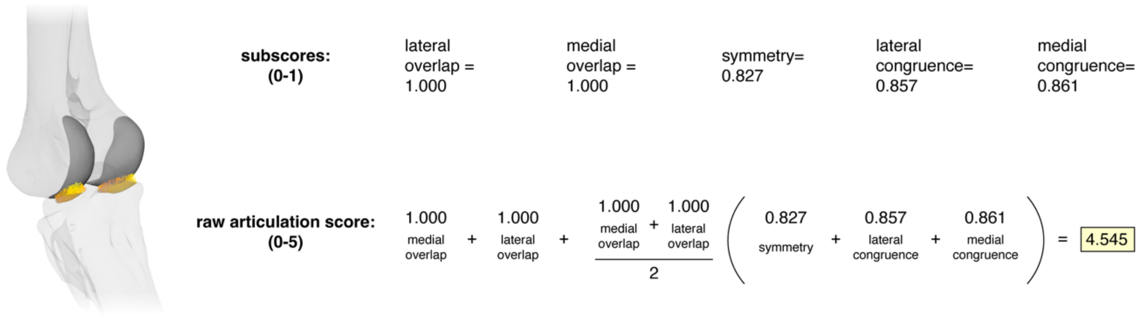

Supplementary Figure 15. Example of calculation of raw articulation score given overlap, symmetry, and congruence subscores.
