# Supplementary material for: Articular surface interactions distinguish dinosaurian locomotor joint poses
Source: Nat Commun. 2024 Feb 16;15:854. doi: 10.1038/s41467-024-44832-z (PMC10873393; doi:10.1038/s41467-024-44832-z)
Supplement: Supplementary file 3 — Description of Additional Supplementary Files [file 41467_2024_44832_MOESM3_ESM.pdf]

Supplementary Data 1. Morphologically informed translation ranges for articulation analysis. Radii and heights of cylinders fit to distal tibiotarsal (ankle), distal third tarso/metatarsal (metatarsophalangeal [MTP]) condyles, or distal phalangeal (interphalangeal [IP]) condyles, and resulting calculated translation ranges following the prism-based hinge joint translation convention of Manafzadeh & Gatesy (2021), for each individual. All measurements in millimeters, rounded to three decimal places, and converted to right-handed sign conventions for ease of comparison. See also Supplementary Fig. 10.

Supplementary Movie 1. Visualization of articulation analysis framework. See also Fig. 1.

Supplementary Movie 2. Reconstructed stride cycle for *Deinonychus antirrhopus* based on articulation analysis. See also Fig. 3, Supplementary Movie 3-4.

Supplementary Movie 3. Reconstructed stride cycle for *Deinonychus antirrhopus* based on articulation analysis, from additional perspectives. See also Fig. 3, Supplementary Movie 2, 4.

Supplementary Movie 4. Reconstructed stride cycle for *Deinonychus antirrhopus* based on articulation analysis, including articular raycasts. See also Fig. 3, Supplementary Movie 2-3.
