# Supplementary material for: Articular surface interactions distinguish dinosaurian locomotor joint poses
Source: Nat Commun. 2024 Feb 16;15:854. doi: 10.1038/s41467-024-44832-z (PMC10873393; doi:10.1038/s41467-024-44832-z)
Supplement: Supplementary file 9 — Reporting Summary [file 41467_2024_44832_MOESM9_ESM.pdf]

nature portfolio

Corresponding author(s): Armita R. Manafzadeh

Last updated by author(s): Dec 29, 2023

Reporting Summary

Nature Portfolio wishes to improve the reproducibility of the work that we publish. This form provides structure for consistency and transparency in reporting. For further information on Nature Portfolio policies, see our Editorial Policies and the Editorial Policy Checklist.

Statistics

For all statistical analyses, confirm that the following items are present in the figure legend, table legend, main text, or Methods section.

n/a	Confirmed	
☐	☒	The exact sample size (*n*) for each experimental group/condition, given as a discrete number and unit of measurement
☒	☐	A statement on whether measurements were taken from distinct samples or whether the same sample was measured repeatedly
☒	☐	The statistical test(s) used AND whether they are one- or two-sided<br>*Only common tests should be described solely by name; describe more complex techniques in the Methods section.*
☒	☐	A description of all covariates tested
☒	☐	A description of any assumptions or corrections, such as tests of normality and adjustment for multiple comparisons
☒	☐	A full description of the statistical parameters including central tendency (e.g. means) or other basic estimates (e.g. regression coefficient) AND variation (e.g. standard deviation) or associated estimates of uncertainty (e.g. confidence intervals)
☒	☐	For null hypothesis testing, the test statistic (e.g. *F*, *t*, *r*) with confidence intervals, effect sizes, degrees of freedom and *P* value noted<br>*Give P values as exact values whenever suitable.*
☒	☐	For Bayesian analysis, information on the choice of priors and Markov chain Monte Carlo settings
☒	☐	For hierarchical and complex designs, identification of the appropriate level for tests and full reporting of outcomes
☒	☐	Estimates of effect sizes (e.g. Cohen's *d*, Pearson's *r*), indicating how they were calculated

Our web collection on statistics for biologists contains articles on many of the points above.

Software and code

Policy information about availability of computer code

Data collection	Native functions within XMALab Version 2.1.0 and Autodesk Maya Version 2023 were used to collect data (no custom code).
Data analysis	Pre-existing custom code within Autodesk Maya Version 2023 and native Maya functions were used to analyze data. Code available at https://bitbucket.org/xromm/workspace/projects/XROMM.

For manuscripts utilizing custom algorithms or software that are central to the research but not yet described in published literature, software must be made available to editors and reviewers. We strongly encourage code deposition in a community repository (e.g. GitHub). See the Nature Portfolio guidelines for submitting code & software for further information.

Data

Policy information about availability of data

All manuscripts must include a data availability statement. This statement should provide the following information, where applicable:

- Accession codes, unique identifiers, or web links for publicly available datasets
- A description of any restrictions on data availability
- For clinical datasets or third party data, please ensure that the statement adheres to our policy

Guineafowl CT scans and associated in vivo XROMM data have been previously published by Manafzadeh et al. (2021) and are available on the XMAPortal at https://xmaportal.org/webportal/larequest.php?request=CollectionView&StudyID=20&instit=BROWN&collectionID=15; emu CT scans were provided by John R. Hutchinson and are available on request directly from him. 3D meshes used to demonstrate the potential for broader application of articulation analysis in the Supplementary

Information are available in the National Institutes of Health 3D Portal at https://3d.nih.gov/entries/3DPX-000387 and the Harvard Dataverse https://doi.org/10.7910/DVN/XP3JVZ. 3D meshes of the Deinonychus antirrhopus pedal elements studied here are available through MorphoSource at https://www.morphosource.org/projects/000592906?locale=en or on request from the Yale Peabody Museum of Natural History.

Research involving human participants, their data, or biological material

Policy information about studies with human participants or human data. See also policy information about sex, gender (identity/presentation), and sexual orientation and race, ethnicity and racism.

Reporting on sex and gender	N/A
Reporting on race, ethnicity, or other socially relevant groupings	N/A
Population characteristics	N/A
Recruitment	N/A
Ethics oversight	N/A

Note that full information on the approval of the study protocol must also be provided in the manuscript.

Field-specific reporting

Please select the one below that is the best fit for your research. If you are not sure, read the appropriate sections before making your selection.

☒ Life sciences ☐ Behavioural & social sciences ☐ Ecological, evolutionary & environmental sciences

For a reference copy of the document with all sections, see nature.com/documents/nr-reporting-summary-flat.pdf

Life sciences study design

All studies must disclose on these points even when the disclosure is negative.

Sample size	In vivo XROMM data were collected from five ankles across four individuals, as discussed by and reported in Manafzadeh et al. (2021) PNAS. Three guineafowl ankles were tested to confirm reproducibility of the analyses (see Supplementary Information) and then one of each joint was used for the remainder of the study.
Data exclusions	No data were excluded.
Replication	One set of sensitivity analyses was conducted (see Supplementary Information) to confirm reproducibility of results and was successful.
Randomization	N/A; this study did not involve experimental groups.
Blinding	N/A; this study did not involve experimental groups.

Reporting for specific materials, systems and methods

We require information from authors about some types of materials, experimental systems and methods used in many studies. Here, indicate whether each material, system or method listed is relevant to your study. If you are not sure if a list item applies to your research, read the appropriate section before selecting a response.

Materials & experimental systems

n/a	Involved in the study	
☒	☐	Antibodies
☒	☐	Eukaryotic cell lines
☐	☒	Palaeontology and archaeology
☐	☒	Animals and other organisms
☒	☐	Clinical data
☒	☐	Dual use research of concern
☒	☐	Plants

Methods

n/a	Involved in the study	
☒	☐	ChIP-seq
☒	☐	Flow cytometry
☒	☐	MRI-based neuroimaging

Palaeontology and Archaeology

Specimen provenance	N/A; the Deinonychus antirrhopus specimen used in this study is an existing specimen housed at the Yale Peabody Museum; YPM 5205.
Specimen deposition	The Deinonychus antirrhopus specimen used in this study is housed at the Yale Peabody Museum; YPM 5205.
Dating methods	N/A; the Deinonychus antirrhopus specimen used in this study is an existing specimen housed at the Yale Peabody Museum; YPM 5205.

☐ Tick this box to confirm that the raw and calibrated dates are available in the paper or in Supplementary Information.

Ethics oversight	N/A

Note that full information on the approval of the study protocol must also be provided in the manuscript.

Animals and other research organisms

Policy information about studies involving animals; ARRIVE guidelines recommended for reporting animal research, and Sex and Gender in Research

Laboratory animals	All guineafowl (Numida meleagris) in vivo data reported here was originally reported by Manafzadeh et al. (2021) PNAS; see that publication for full information.
Wild animals	N/A
Reporting on sex	N/A
Field-collected samples	N/A
Ethics oversight	All procedures conducted with live animals were approved by the Brown University Institutional Animal Care and Use Committee; see Manafzadeh et al. (2021) PNAS.

Note that full information on the approval of the study protocol must also be provided in the manuscript.
